# Exogenous hydrogen peroxide increases dry matter production, mineral content and level of osmotic solutes in young maize leaves and alleviates deleterious effects of copper stress

**DOI:** 10.1186/1999-3110-54-26

**Published:** 2013-08-30

**Authors:** Sule Guzel, Rabiye Terzi

**Affiliations:** 1 Department of Biology, Faculty of Arts and Sciences, Recep Tayyip Erdoğan University, Rize, 53100 Turkey; 2 Department of Biology, Faculty of Sciences, Karadeniz Technical University, Trabzon, 61080 Turkey

**Keywords:** Copper toxicity, H_2_O_2_ treatment, Maize, Mineral uptake, Osmotic solute

## Abstract

**Background:**

The effects of exogenously applied H_2_O_2_ on growth, water status, the mineral ion content (Na^+^, K^+^, Ca^2+^, Mg^2+^ and Cu^2+^), proline, total sugars and soluble proteins were assessed in leaves of maize (*Zea mays* L.) cultivars, Akpinar and Pegaso exposed to excess copper (0.5 mM). Seedlings were grown in equal-sizes plastic pots and irrigated with Hoagland nutrient solution containing H_2_O_2_ or/and copper. Different treatments taken for pot experiments were named as the control (C), H_2_O_2_ treatment only (H_2_O_2_), excess Cu (Cu) and, Cu stress combined with H_2_O_2_ pretreatment (Cu + H_2_O_2_).

**Results:**

Treatment of H_2_O_2_ caused the increases in growth, water content, mineral concentration, proline, total sugar and soluble protein contents compared to the control groups in the leaves of both cultivars. Yet excess copper caused reductions in the growth, leaf water potential, Na^+^, K^+^, Ca^+^, Mg^2+^ concentrations and soluble protein levels but increases in proline, total soluble sugars and Cu^2+^ contents compared to the control group. Dry matter, leaf water potential and mineral content of Cu + H_2_O_2_ group revealed a lower decrease than Cu group ones. A higher increase was also observed in proline and total sugar contents of Cu + H_2_O_2_ group than Cu group ones in both cultivars.

**Conclusions:**

These data revealed that exogenous H_2_O_2_ might increase the dry matter production and the mineral ion distribution in maize seedlings. Moreover, osmotic regulation might be involved in alleviation of copper toxicity of maize leaves by pretreatment of H_2_O_2_.

**Electronic supplementary material:**

The online version of this article (doi:10.1186/1999-3110-54-26) contains supplementary material, which is available to authorized users.

## Background

Modern human activities like mining, smelting, municipal waste disposal and electroplating are the important sources of heavy metal pollution in the environment. Among the heavy metals, copper (Cu^2+^) is considered to be one of the most important pollutants of the air and is also very significant pollutant of agricultural soils (Alloway, 1990). Soils may contain elevated levels of Cu because of its widespread use as a pesticide, land application of sewage sludge as well as mining and smelting activities. However, Cu is essential micronutrient for normal plant metabolism and involved in a number of physiological processes such as photosynthesis, respiration, carbohydrate distribution and protein metabolism. On the other hand, even metabolic metals beyond a threshold concentration are harmful to biological systems (Foy et al., 1978). For instance, excess Cu might decrease the leaf water content, shoot elongation, plant biomass and germination rate (Ahsan et al., 2007).

Modulation of water content, biomass and osmotic solutes in plants after exposure to copper was frequently reported in the literature (*e.g.*, 2001). Important among a variety of compatible osmolytes in higher plants were soluble sugars, organic acids, proline, soluble proteins (Ashraf, 2004) and inorganic minerals such as sodium, potassium, calcium and chlorine (Chen and Jiang, 2010). Sucrose content, one of soluble sugars increased in the cucumber leaves by copper stress and the uptake and the upward translocation of the ions were altered (Alaoui-Sosse et al., 2004). Similarly, proline content increased in plants when exposed to wide variety of environmental stresses and thus provided the plants protection against damage by reactive oxygen species (Aspinall and Paleg, 1981). For instance, excess copper induced accumulation of proline in various plants such as *Oryza sativa* (Chen et al., 2001) and *Nicotiana benthamiana* (Ku et al., 2012). Adversely, it has been shown a decrease in soluble protein level at several stress condition like copper and aluminum stresses (Yadav et al., 2009). Namely, both organic and inorganic solutes are essential for osmoregulation in plants, especially stress conditions. However, their relative contribution to osmotic adjustment varies from plant to plant or species to species, or even within different tissue of same plant (Ashraf, 2004). On the other hand, it has been well known that H_2_O_2_ is a major kind of reactive oxygen species in plant tissues. However, increasing lines of evidence supported the idea that H_2_O_2_ might act as a signal molecule with multiple functions in plants. Numerous studies have shown that the application of H_2_O_2_ at low concentrations could improve plant tolerance to heat stress (Gao et al., 2010), salt stress (De Azevedo Neto et al., 2005; Li et al. 2011), aluminum-induced oxidative stress (Xu et al., 2011) and heavy metal stress (Lin et al. 2004; Hu et al., 2009). The increased metal stress tolerance was attributed to induced antioxidant defense system after pretreatment with H_2_O_2_ in rice seedlings (Hu et al., 2009). Despite a large number of reports detailing plant tolerance mechanisms to heavy metals, plant responses to H_2_O_2_ pretreatment under heavy metal toxicity are not properly understood.

It is known that crop plants, such as maize, wheat and barley may be subjected to heavy metal stress during their growing period. The accumulation of heavy metals in the seeds and other above ground parts of maize has been becoming a serious problem for agriculture and human health. Maize (*Zea mays* L.) is an important staple C4 food crop in many countries of the world and it accounts for around 712 million metric tones in 2006. Early studies showed that excess copper reduced plant biomass and cell increment of many plant species (Ouzounidou et al., 1998). As mentioned, application of H_2_O_2_ at low concentrations could induce stress tolerance in plants. However, the effect of H_2_O_2_ pretreatment to Cu toxicity is far from unclear. The objective of this study was to determine the response of H_2_O_2_ pretreatment to high copper content in two maize cultivars, Akpinar and Pegaso differently sensitive to water deficit. The biomass accumulation, alterations in water status, mineral ion content (Na^+^, K^+^, Ca^2+^ Mg^2+^ and Cu^2+^), soluble protein, proline and total soluble sugars were investigated in the leaves of two maize cultivars pretreated with H_2_O_2_ under Cu stress.

## Methods

### Plant material, growth and H_2_O_2_/CuSO_4_ treatment

Two *Zea mays* L. cultivars differently sensitive to water deficit (cv. Pegaso more tolerant than Akpinar cv.) were provided from Black Sea Agricultural Research Institute, Eskisehir, Turkey and Limagrain Seed Production Company, Istanbul, Turkey. Seeds were surface sterilized with 0.1% HgCl_2_ for 3 min followed by repeated washings with sterilized distilled water. Seeds were sown in pots (14 cm height, 16 cm top and 11 cm bottom diameter) containing sterilize water washed sand. Seedlings were grown in a growth chamber with the following parameters: 16 h light and 8 h darkness at 25°C ± 2, relative humidity 60% ± 5, photon flux density of at the surface of the leaves 400 μmol m^-2^ s^-1^ and were watered once per two days with Hoagland nutrient solution (pH 6.0). When the second leaves were fully expanded, the pots were divided into four groups for each maize cultivar. Two groups were watered with Hoagland nutrient solution, and the other two groups were watered with freshly prepared Hoagland nutrient solution containing 0.5 mM H_2_O_2_. The H_2_O_2_-applied plants were watered with either the Hoagland nutrient solution only or Hoagland nutrient solution containing 0.5 mM CuSO_4_ for additional five days and were designated as “H_2_O_2_ treatment (H_2_O_2_)”, and “Cu + H_2_O_2_ pretreatment (Cu + H_2_O_2_)”, respectively. The other two groups of the pots were separately watered with either of the two types of nutrient solutions and were named “control (C),” and Cu stress (Cu)”. After two days of the treatments, samples of the third leaf were used for the following analyses.

### Cu analysis

Leaf samples were extensively washed with distilled water and then the samples were oven-dried and ground to power. The powder was ashen in a muffle furnace at 550°C for 4 h and the residue was brought to a standard volume with 1 M HNO_3_. Cu concentration of the extract was determined by a flame atomic absorbption spectrophotometer (Unicam, 929 AAS). The copper content of tissues was expressed as μg/g dry weight.

### Determination of hydrogen peroxide content

To determine endogenic H_2_O_2_ content after exogenous applications, hydrogen peroxide content was determined according to modified method of Velikova et al. (2000). Leaves (0.25 g) were ground in 3 ml of 5% trichloroacetic acid with 0.1 g activated charcoal at 0°C. To 0.5 ml aliquot of the supernatant, 0.5 ml of 10 mM potassium phosphate buffer (pH 7.0) and 0.75 ml of 1 M KI were added. The absorbance was measured at 390 nm and H_2_O_2_ content was expressed as μmol g^-1^ fresh weight.

### Growth measurement

To obtain enough data on variations in the growth of the plants, the measurement of dry weight, and the grade of growth inhibition (GGI) were recorded. To measure dry weight, the plants were harvested from four group pots and fresh weights of the leaves were determined. Then the samples were oven-dried at 80°C for 24 hours (Jiang et al., 2004, 1993. All treatments were compared to the control group (control GGI = 0, i.e., 100% growth).

$$\mathrm{Grade}\;\mathrm{of}\;\mathrm{Growth}\;\mathrm{Inhibition}\;\left(\mathrm{GGI}\right)=\;\left[\left(\mathrm{Dry}\;\mathrm{weight}\;\mathrm{of}\;\mathrm{control}\;\mathrm{plants}‒\mathrm{Dry}\;\mathrm{weight}\;\mathrm{of}\;\mathrm{treated}\;\mathrm{plants}\right)/\left(\mathrm{Dry}\;\mathrm{weight}\;\mathrm{of}\;\mathrm{control}\;\mathrm{plants}\right)\right]\times 100$$

### Measurement of water content

Leaf water potential (Ψ_leaf_) was measured with a C52 thermocouple psychrometer (Wescor, Inc., Logan, UT, USA). Discs about 6 mm in diameter were cut from leaves and sealed in a C-52 psychrometer chamber. The readings were recorded by a Wescor PSYPRO water potential data logger in the psychrometric mode after samples were equilibrated for 45 min. Values of leaf water potential were measured as MPa.

Relative water content (RWC) of the leaves was estimated according to the method of Castillo (Castillo, 1996). Samples (0.5 g) were saturated in 100 ml of distilled water for 24 h at 4°C in the dark and their turgid weights were recorded. Subsequently, they were dried at 65°C of oven for 48 h and their dry weights were recorded. RWC was calculated as given below:

$$\mathrm{RWC}\left(\%\right)=\left[\left(\mathrm{FW}‒\mathrm{DW}\right)/\left(\mathrm{TW}‒\mathrm{DW}\right)\right]\times 100$$

where FW, DW, and TW are fresh weight, dry weight, and turgid weight, respectively.

### Determination of proline content

Proline content was achieved according to the method Bates et al. (1973), and dried ground leaves (0.25 g) were used for proline extraction. Samples were homogenized in 5 ml 3% sulfosalicylic acid and extracts were centrifuged at 8000 *x g* for 15 min. 1 ml filtrate was mixed with equal volumes of acetic acid and ninhydrin reagent (1.25 g ninhydrin, 30 ml of glacial acetic acid, 20 ml 6 M H_3_PO_4_) and incubated for 1 h at 100°C. The reaction was stopped by placing the test tubes in ice cold water. The samples were rigorously mixed with 3 ml toluene. After 50 min, the light absorption of the toluene phase was estimated at 520 nm on a UV-VIS spectrophotometer. The proline concentration was determined using a standard curve. Free proline content was expressed as μg/g dry weight.

### Determination of soluble protein content

For extraction of soluble proteins, the leaf samples were freshly harvested and fixed in liquid nitrogen. Thawed leaves were homogenized in 0.05 M sodium phosphate buffer (pH 6.0) with 0.1% polyvinylpolypyrrolidone (PVPP). The homogenate was filtered through cheesecloth. The filtered homogenate was centrifuged at 20000 *x g* for 20 min at 4°C. After centrifugation, the supernatant was taken and used for protein measurements. Soluble protein content was assayed according to Bradford (1976). Absorbance was read on a UV-VIS spectrophotometer at 595 nm. The soluble protein concentration was determined by using bovine serum protein (BSA, Sigma, USA) as a standard and expressed as mg/g fresh weight.

### Determination of total soluble sugar content

Total soluble sugar content was determined by phenol-sulfiric acid method (Dubois et al., 1956). Dry leaves (0.1 g) were extracted with 5 ml of 80% ethanol, by boiling the samples in glass tubes in a 95°C-water bath for 10 min. After extraction, the tubes were centrifuged at 489 *x g* for 5 min, and the supernatants of the extractions were used for sugar analysis. One hundred ml of sample was added to 900 ml of distilled water then mixture was vortexed. One ml of 5% phenol and 5 ml of H_2_SO_4_ were added to 1 ml of sample and the mixture was stirred. After cooling under room temperature for 15 min, absorbance of sample was recorded at 490 nm.

### Analysis of mineral ions

Total ion content (Na^+^, Ca^2+^, K^+^ and Mg^2+^) was measured with a pH/mV/Ion/Temp meter (JENCO 6251 N). The leaf samples (0.5 g) were homogenized with liquid nitrogen in 5 ml deionized water. The homogenate was boiled in a water bath for 10 min. The precipitate was removed by centrifugation.

### Statistical analysis

Analysis of variance (ANOVA) of means of 6 replicates for leaf water potential, RWC, dry weight, amounts of inorganic ions, proline, protein and total soluble sugar contents was performed with Duncan Multiple Comparison test using SPSS for Microsoft Windows (Ver. 10.0, SPSS Inc., USA) and statistical significance between the two cultivars along with different treatments was determined at P < 0.05 level.

## Results

### Copper content

**Figure 1:**
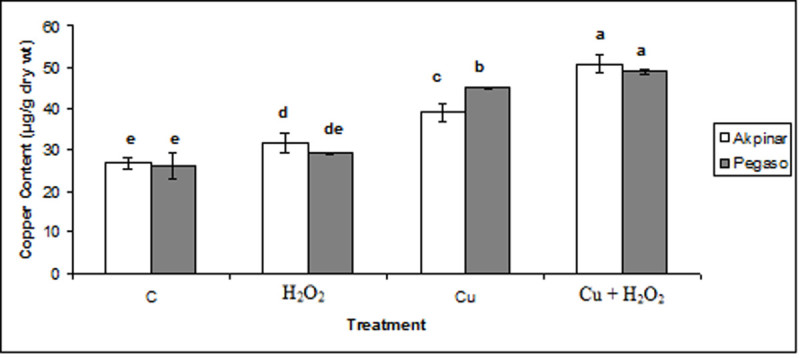
**The effect of H**_**2**_**O**_**2**_**application on Cu concentration under excess copper in maize cultivars.** The plants were submitted to four treatments: control (C); H_2_O_2_ treatment (H_2_O_2_); Cu stressed (Cu); Cu stressed plus H_2_O_2_ pretreatment (Cu + H_2_O_2_). Vertical bars represent standard deviation. Different letters denote significant differences between the two cultivars along with different treatments at *p* < 0.05.

Copper concentrations were determined in the leaves of the test plants and shown in Figure 1. The plants that grown under 0.5 mM Cu revealed high Cu concentration. Exogenous H_2_O_2_ applications (H_2_O_2_ group and Cu + H_2_O_2_ group) also caused the increases in the copper content of the leaves of both cultivars. However, the increases in Akpinar cv. were higher than Pegaso cv. ones in both H_2_O_2_ group and Cu + H_2_O_2_ group. For instance, in Pegaso cv., Cu concentrations were measured as 26 ± 3, and 29 ± 0.4 μg g^-1^ dry weight in the control and H_2_O_2_-treated group, respectively. Under excess Cu applications, Cu concentrations were recorded as 45 ± 0.3 and 49 ± 0.4 μg g^-1^ dry weight in the copper exposed plants and copper stress plus H_2_O_2_ pretreated plants, respectively. As for Akpinar cv., Cu concentrations were found as 27 ± 2, and 32 ± 2 μg g^-1^ dry weight in the control and H_2_O_2_-treated group, respectively. Also, under excess Cu applications, the concentrations were recorded as 39 ± 2 and 51 ± 2 μg g^-1^ dry weight in the Cu group and Cu + H_2_O_2_ group, respectively (Figure 1).

### H_2_O_2_ content

**Figure 2:**
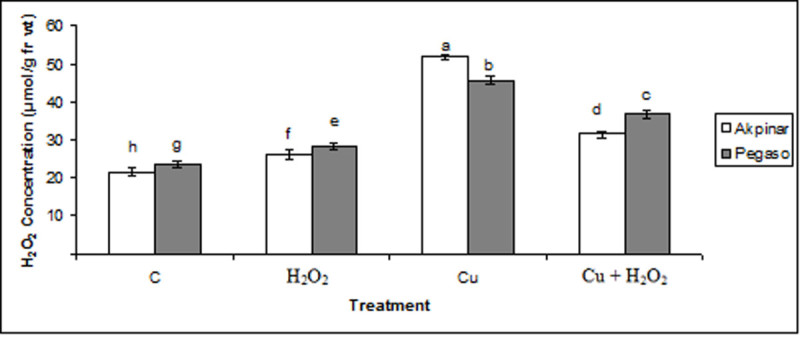
**The effect of H**_**2**_**O**_**2**_**application on H**_**2**_**O**_**2**_**content under excess copper in maize cultivars.** Vertical bars represent standard deviation. Different letters denote significant differences between the two cultivars along with different treatments at *p* < 0.05.

Endogenic H_2_O_2_ content after exogenous applications increased in two cultivars. Also, the H_2_O_2_ concentration significantly increased under excess copper. Pretreatment with H_2_O_2_ also reduced the overproduction of H_2_O_2_ in both cultivars under copper stress (Figure 2).

### Plant growth

**Figure 3:**
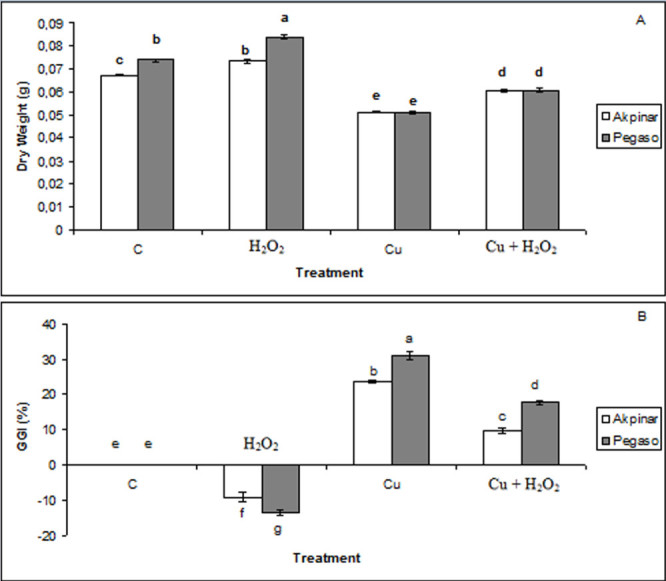
**The effect of H**_**2**_**O**_**2**_**application on dry weight and GGI (%) under excess copper in maize cultivars. A** and **B** represent dry weight and GGI (%), respectively. Vertical bars represent standard deviation. Different letters denote significant differences between the two cultivars along with different treatments at *p* < 0.05.

Toxicity of copper and the effect of H_2_O_2_ application on the growth of maize cultivars, Akpinar and Pegaso are presented in Figure 3. To understand how H_2_O_2_ induced tolerance to copper stress, we studied the changes in dry matter and mineral ion content, and osmoregulation mechanism of the leaves of two maize cultivars. While maize cultivars exposure to excess Cu showed a significant decrease in dry matter, dry weights of H_2_O_2_-treated plants revealed a significant increase. H_2_O_2_ pretreatment also reduced the decrease in amount of dry matter in both cultivars (Figure 3 A).

A protocol used for phytotoxicity testing, the grade of growth inhibition (%) was also evaluated in this study and a significant GGI (%) was observed in plants treated with 0.5 mM Cu. However, the copper-induced growth inhibition decreased when the seedlings were pretreated with H_2_O_2_. GGI values were recorded as -9% (negative value represents the increase in growth), 24%, and 10% in H_2_O_2_ group, Cu group and Cu + H_2_O_2_ group of Akpinar cultivar, respectively. As for Pegaso, the values were found as -14%, 31% and 18% in H_2_O_2_ group, Cu group and Cu + H_2_O_2_ group, respectively (Figure 3 B).

### Water status

**Figure 4:**
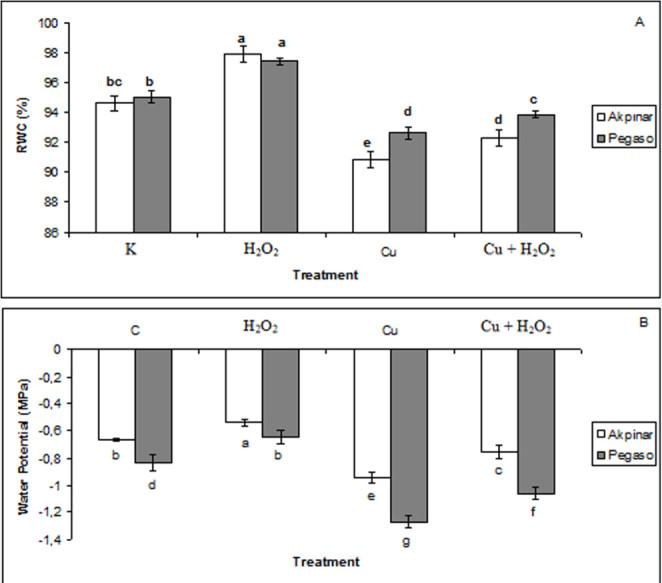
**The effect of H**_**2**_**O**_**2**_**application on leaf water status under excess copper in maize cultivars. A** and **B** represent relative water content (RWC) and leaf water potential (Ψ_leaf_), respectively. Vertical bars represent standard deviation. Different letters denote significant differences between the two cultivars along with different treatments at *p* < 0.05.

In general, decrease in leaf water potential or RWC in plants is considered as direct indicator of the stress. Ψ_leaf_ and RWCs of both maize cultivars statistically decreased in copper stress applied plants compared to control plants. On the contrary, pretreatment of H_2_O_2_ (Cu + H_2_O_2_ group) prevented the decrease in leaf water potential and RWC values. H_2_O_2_ treatment also increased leaf water potential and RWC in both Pegaso and Akpinar cultivars. For instance, while in control plants of Pegaso, Ψ_leaf_ was found as -0.84 MPa, the value was -1.27 MPa in copper stress exposed plants. As mentioned, H_2_O_2_ treatments increased the values of Ψ_leaf_. The values of Ψl_eaf_ were determined as -0.64 MPa and -1.06 MPa in H_2_O_2_ treated plants and copper stress plus H_2_O_2_ pretreated plants, respectively. Similarly, in control plants of Pegaso cultivar, the value of RWC was measured as 95%. The value was 92.6% under excess copper. However, RWC values were estimated as 97.4% and 93.8%, in H_2_O_2_ treated plants and copper stress plus H_2_O_2_ pretreated plants, respectively. A similar trend of the Ψ_leaf_ and RWC was also recorded in Akpinar cultivar (Figure 4).

### Proline content

**Figure 5:**
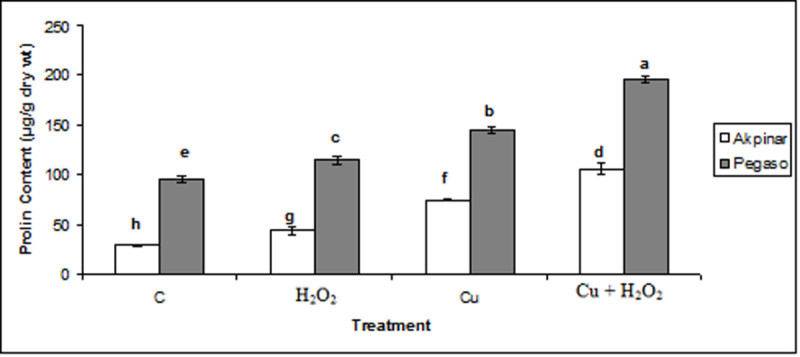
**The effect of H**_**2**_**O**_**2**_**application on proline content under excess copper in maize cultivars.** Vertical bars represent standard deviation. Different letters denote significant differences between the two cultivars along with different treatments at *p* < 0.05.

The proline content of the maize cultivars exposed to Cu or/and H_2_O_2_ exhibited a specific increase. Exposure to either copper or H_2_O_2_ resulted in a rise in proline level of test plants. Interestingly, in unstressed plants, treatment of H_2_O_2_ also increased the proline level. The proline accumulation was of the greatest magnitude in copper stress plus H_2_O_2_ pretreatment (Cu + H_2_O_2_) (Figure 5).

### Soluble protein content

**Figure 6:**
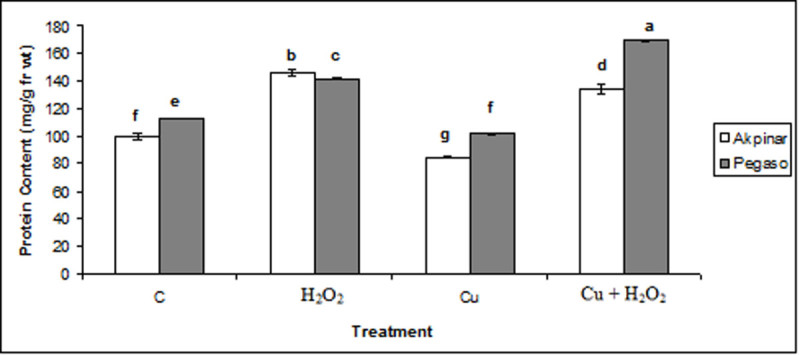
**The effect of H**_**2**_**O**_**2**_**application on soluble protein content under excess copper in maize cultivars.** Vertical bars represent standard deviation. Different letters denote significant differences between the two cultivars along with different treatments at *p* < 0.05.

The results of Figure 6 showed that soluble protein contents of Pegaso and Akpinar cultivars decreased in plants exposed to excess copper, however, H_2_O_2_ applications prevented the decrease in soluble protein content. Moreover, treatment with H_2_O_2_ alone increased the protein content in both cultivars. Similarly, protein content increased in the copper stress plus H_2_O_2_ pretreatment (Cu + H_2_O_2_) compared to control group.

### Total soluble sugars

**Figure 7:**
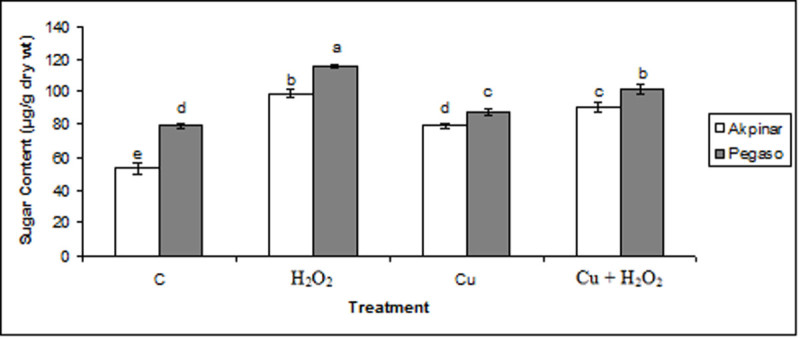
**The effect of H**_**2**_**O**_**2**_**application on total soluble sugar under excess copper in maize cultivars.** Vertical bars represent standard deviation. Different letters denote significant differences between the two cultivars along with different treatments at *p* < 0.05.

The total soluble sugar contents of the control plants were measured as 53 ± 0.004 μg and 79 ± 0.001 μg per g dry weight in Akpinar and Pegaso cultivars, respectively. After treatment with H_2_O_2_ (H_2_O_2_ group), total soluble sugar contents in the leaves were elevated in both cultivars. The Cu exposure (Cu group) also increased the total soluble sugar content significantly in the leaves of maize cultivars compared to the control group. The soluble sugar content showed a higher increase under pretreatment of H_2_O_2_ (Cu + H_2_O_2_ group) than under Cu stress (Figure 7).

### Inorganic ion concentrations

**Figure 8:**
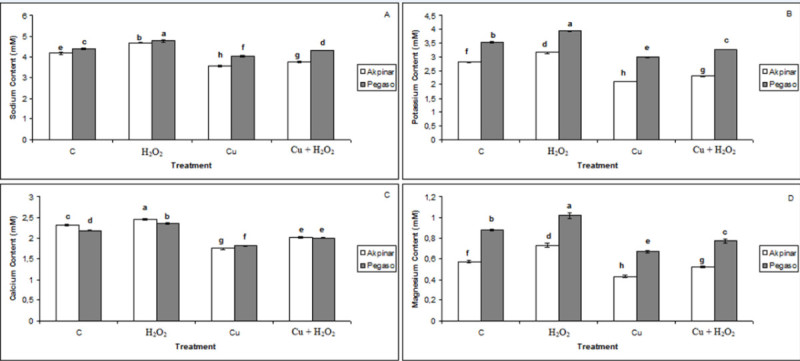
**The effect of H**_**2**_**O**_**2**_**application on inorganic ions under excess copper in maize cultivars. A:** Sodium content, **B:** Potassium content, **C:** Calcium content, **D:** Magnesium content. Vertical bars represent standard deviation. Different letters denote significant differences between the two cultivars along with different treatments at *p* < 0.05.

In Figure 8 the results for ion concentration of the leaves have been presented, for these elements where significant changes were detected. Ion concentrations (Na^+^, Ca^2+^, K^+^ and Mg^2+^) of the leaves of maize cultivars showed that the content of these ions was decreased by excess copper (Cu group) but increased by H_2_O_2_ treatment (H_2_O_2_ group). Mineral ion concentration of the H_2_O_2_-treated maize cultivars was the highest. When H_2_O_2_ pretreatment was combined with the excess Cu (Cu + H_2_O_2_ group), the decrease in the content of these ions was prevented slightly.

## Discussion

In recent years, it has been reported that H_2_O_2_ pretreatment alleviates the damages of the some abiotic stresses such as salt stress in wheat seedlings (Li et al., 2011), oxidative stress in young pea leaves (Moskova et al., 2009), heat stress in cucumber leaves (Gao et al., 2010), aluminum-induced oxidative stress in wheat seedlings (Xu et al., 2011) and cadmium stress in rice seedlings (Hu et al., 2009). However, the exact mechanism of the protective action of H_2_O_2_ application at low concentrations against various stresses especially copper stress is not yet elucidated. In the present study, we investigated whether exogenous H_2_O_2_ could protect the growth and water content under excess copper and whether the protective effect was associated with the nutrient concentration and osmotic regulation in the leaf tissues. We determined that treatment of maize cultivars with excess copper resulted in a sharply decrease in dry matter of the leaves but exogenous H_2_O_2_ applications improved the amount of dry matter. Similar to our data, Mocquot et al. (1996), reported a significant decrease in root and leaf biomass of 14-days old maize leaves at 10 μM Cu concentration. Khandaker et al. (2012), also recorded that exogenous H_2_O_2_ increased photosynthetic rates and dry matter content of the leaves in wax apple under field conditions. Collectively, the dry matter enhancement by exogenous application of H_2_O_2_ may be of tremendous agricultural importance especially in copper contaminated area. We also observed that exogenous H_2_O_2_ prevented the grade of growth inhibition in maize cultivars under excess copper. Our findings suggested that H_2_O_2_ application alleviated the hazardous effect of the copper metals on the plant growth. Accordingly, De Azevedo Neto et al. (2005), observed that salt-induced growth inhibition decreased when the seedlings were pretreated with H_2_O_2_.

Exposure to heavy metals including Cu is known to deteriorate the plant water balance. The present investigation also showed that pretreatment of H_2_O_2_ protected the water content of the maize leaves under copper stress. Similar to our results, Wahid et al. (2007), reported that H_2_O_2_ treatment improved leaf water relations of salinity-treated wheat seedlings by turgor maintenance. Liao et al. (2012) also proposed that H_2_O_2_ in vase solution of cut flowers reduced the decline in leaf relative water content.

In the present study, it was also researched the changes in substances involved in osmotic regulation such as proline, soluble protein, total sugar and some mineral ions. As known, the proteinogenic amino acid proline functions as an osmolyte, radical scavenger, electron sink, stabilizer of macromolecules, and a cell wall component (Aspinall and Paleg, 1981). Proline accumulation as a function of excess metal was observed in Pegaso and Akpinar cultivars. The increased proline content in maize cultivars exposed to copper stress may be associated with the response to water deficit. Indeed, excess Cu-induced accumulation of proline has been recorded in various plants such as *Oryza sativa* (Chen et al., 2001) and *Nicotiana benthamiana* (Ku et al., 2012). We also observed that proline accumulation was high in the plants treated with H_2_O_2_ in two maize cultivars. Based on its known properties, the increases in H_2_O_2_-treated groups may be ascribed in scavenging of free radicals. In copper stress plus H_2_O_2_ pretreated plants, the increases in proline content also may be attribute to its a variety of functions *e.g.* osmo-regulation, redox-regulation and metal chelation. Similar to our data, Yang et al. (2009), recorded that exogenous H_2_O_2_ treatment led to a significant accumulation of proline in coleoptiles and radicles of maize seedlings.

One of the causes of proline accumulation in plant tissues has been suggested to be hydrolysis of proteins (Aspinall and Paleg, 1981). In the present study, it was actually observed that soluble protein content decreased in the Cu group plants while increasing proline content. Under biotic and abiotic stress factors causing a reduction of growth, protein synthesis is one of the most negatively affected anabolic processes together with photosynthesis, transport of metabolites, and uptake and translocation of mineral ions (Bonjoch and Tamayo, 2003). On the other hand, it is known that copper ions can readily oxidize the thiol bonds present in the proteins, causing disruption of their structure and functions (Mouratao et al., 2009). Similar to our data, Yadav and Mohanpuria (2009), recorded that protein content decreased after exposure to Cu in the young leaves of two *Camellia sinensis* cultivars. On the other hand, we found that the copper-induced the loss of soluble protein disappeared when the seedlings were pretreated with H_2_O_2_ and moreover, the soluble protein content increased in both maize cultivars compared to the control group. The increased protein content could be due to a maintaining of the structure of proteins and/or could be due to an increased protein synthesis.

As known, sugar accumulation contributes for regulation of internal osmolarity and protection to the biomolecules and membranes (Sinniah et al., 1998). Effect of copper on the total soluble sugar contents of maize cultivars has been presented in Figure 7. These data indicated that excess copper increased the total soluble sugar content in both cultivars. Likewise, sucrose content, one of soluble sugars, enhanced in the cucumber leaves under copper stress (Alaoui-Sosse et al., 2004). We also observed that H_2_O_2_ pretreatment increased the total soluble sugar content in both maize cultivars. In line with these findings, exogenous H_2_O_2_ applications might protect the water status of the tissues by providing osmotic regulation.

We also researched the changes in ion concentrations involved in osmotic adjustment in two maize cultivars under Cu stress. As known, inorganic ions for osmotic adjustment are mainly sodium, calcium, potassium and chlorine (Chen and Jiang, 2010). Our findings showed that Na^+^, Ca^2+^, K^+^ and Mg^2+^ concentrations decreased significantly in Cu-stressed maize cultivars. Accordingly, it was reported a highly significant decreases in Na^+^, Ca^2+^, K^+^ and Mg^2+^ contents under excess copper (Ouzounidou et al., 1998; Alaoui-Sosse et al., 2004). The decline in K^+^ concentration may have played an important role in the inhibition of the growth. Similarly, Alaoui-Sosse et al. (2004), reported that the decrease in K^+^ content may have played a crucial role in the inhibition of leaf expansion. In the same way, a decrease of K^+^ content was observed in rice roots in response to extreme Cu exposure (Chen et al., 2004). As mentioned above, Mg^2+^ concentration decreased as a response to excess copper in both maize cultivars. The reduction in Mg^2+^ concentration may be attributed to the decrease in dry matter of the leaves. Indeed, it was reported that decline of Mg^2+^ concentration could contribute to the reduction in net assimilation rate and to accumulation of assimilates in leaves (Alaoui-Sosse et al., 2004). On the other hand, exogenous H_2_O_2_ applications improved the ion concentrations in both cultivars. According to our results, Wahid et al. (2007), reported that H_2_O_2_-treated wheat seedlings displayed greater tissue Ca^2+^ and K^+^ levels under salt stress. These findings showed that the distribution of the ions could be affected by copper stress but H_2_O_2_ applications might maintain the mineral balance under the stress in maize seedlings. Furthermore, the increases in ion concentrations could be concerned to osmoregulation mechanism.

Maize cultivars exposed to excess Cu accumulated substantial amounts of Cu in their leaves. Exogenous H_2_O_2_ applications also caused the increases in the copper content in both cultivars. The increases make us thinking about that the H_2_O_2_ applications may affect the metal uptake proteins. Indeed, in recent years, it has been recorded that sustained low level of H_2_O_2_ application to cultured cells lead to up-regulation of the major iron uptake protein and thus induced iron uptake (Andriopoulos et al., 2007).

Similar to finding from H_2_O_2_ content studies (*e.g.*, 2011), the present study also showed that H_2_O_2_ contents increased after each treatment in two maize cultivars. H_2_O_2_ is a strong oxidizing agent that injures cells and damages photosynthesis at high concentration when produced internally or applied externally (Wahid et al., 2007). However, H_2_O_2_ pretreatment in sublethal doses can exert a protective effect against copper stress by increasing the levels of osmotic solutes such as ion, proline, soluble protein and sugars in maize cultivars.

## Conclusion

In conclusion, excess copper could inhibit the dry matter production, leaf water content, soluble protein level and mineral ion concentration such as potassium content, an important ion for osmotic regulation, and magnesium content required for photosynthesis, however, pretreatment of H_2_O_2_ could alleviate the hazardous effects of the copper stress. The results indicated that increased proline content and higher level of total soluble sugars might also participate in the alleviation of toxic effect of Cu accumulation. In other words, the changes induced by exogenous H_2_O_2_ in maize metabolism with osmotic adjustment under Cu stress may be involved in the defense of heavy metal. H_2_O_2_ pretreatment might affect the mineral ion distribution including copper in maize seedlings and thus contribute to decreasing of the deleterious effects of copper stress on the growth of maize.

## Authors’ original submitted files for images

Below are the links to the authors’ original submitted files for images. Authors’ original file for figure 1 Authors’ original file for figure 2 Authors’ original file for figure 3 Authors’ original file for figure 4 Authors’ original file for figure 5 Authors’ original file for figure 6 Authors’ original file for figure 7 Authors’ original file for figure 8
