# Supplementary figures and images for: Exogenous hydrogen peroxide increases dry matter production, mineral content and level of osmotic solutes in young maize leaves and alleviates deleterious effects of copper stress
Source: Bot Stud. 2013 Aug 30;54:26. doi: 10.1186/1999-3110-54-26 (PMC5430369; doi:10.1186/1999-3110-54-26)

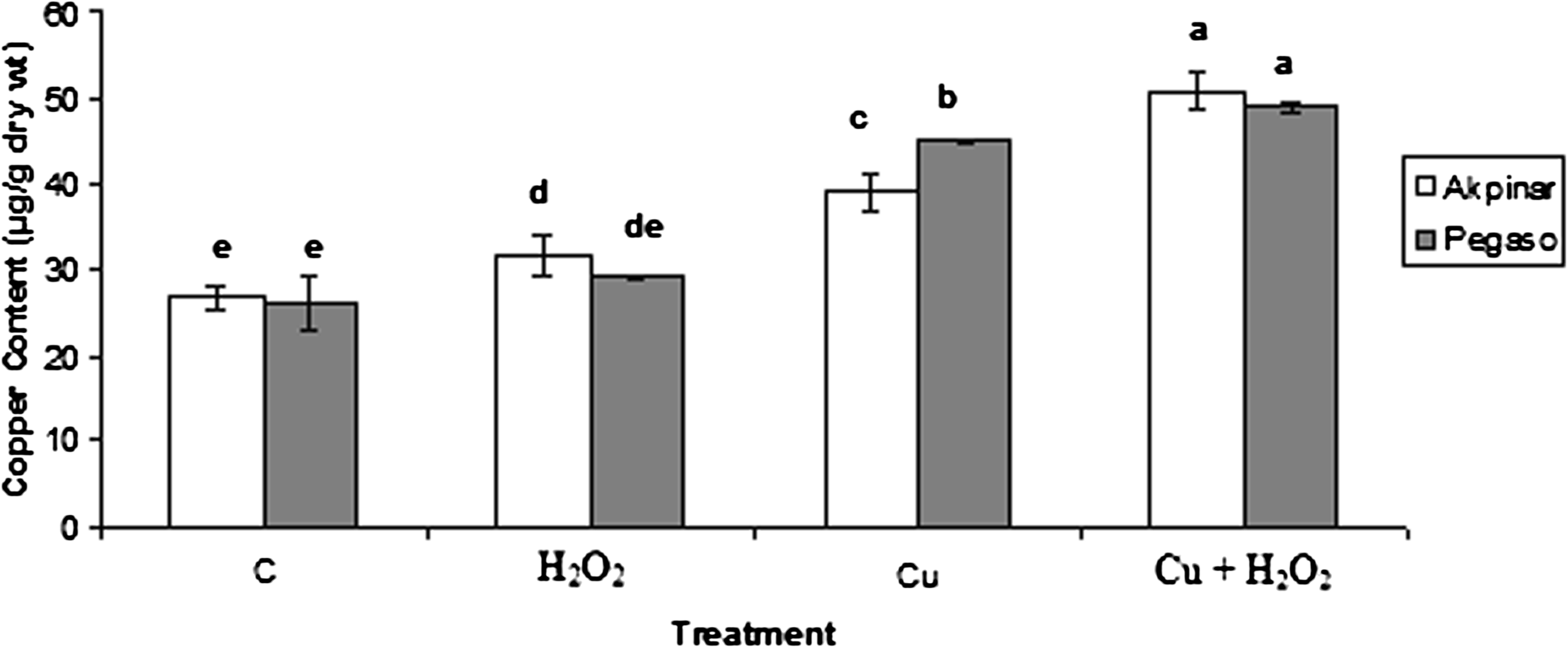

Supplement: Supplementary file 1 — Authors’ original file for figure 1 [file 40529_2012_45_MOESM1_ESM.tif]

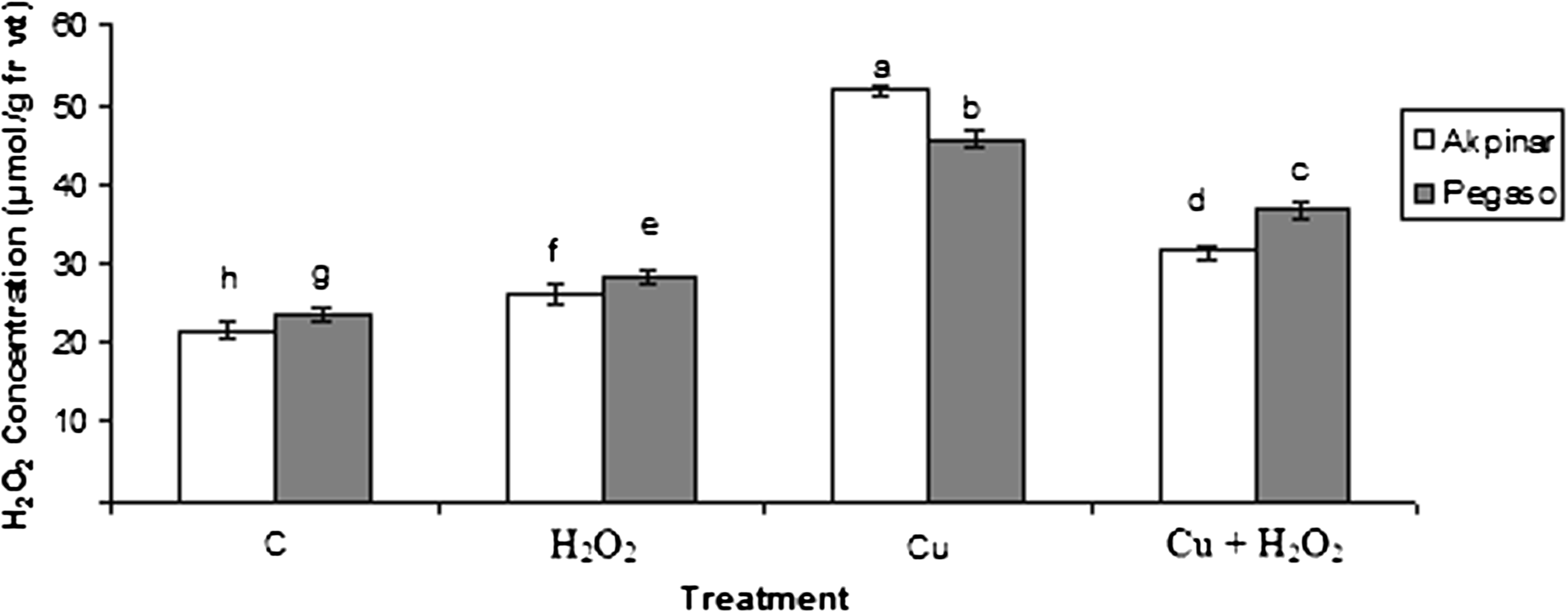

Supplement: Supplementary file 2 — Authors’ original file for figure 2 [file 40529_2012_45_MOESM2_ESM.tif]

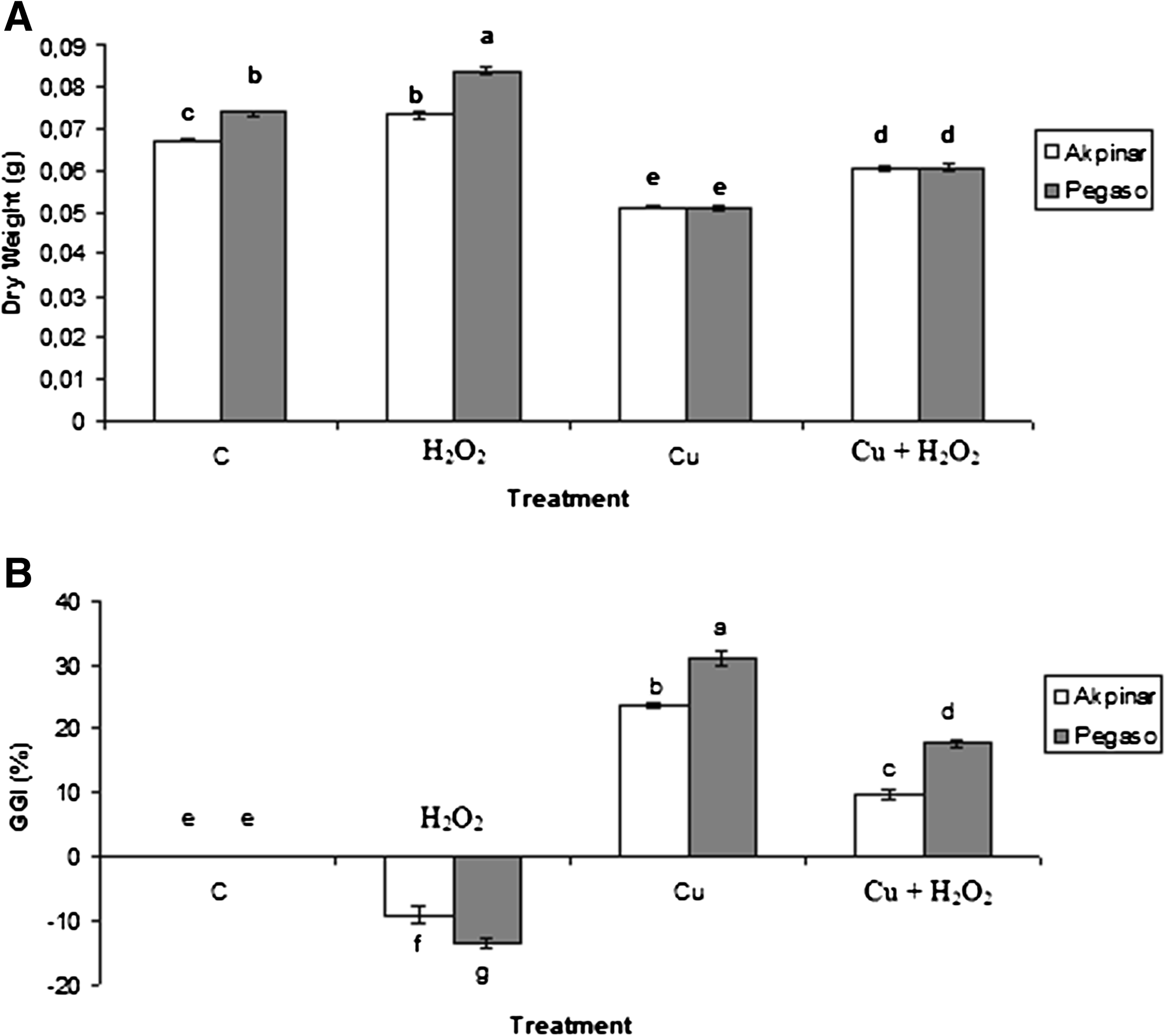

Supplement: Supplementary file 3 — Authors’ original file for figure 3 [file 40529_2012_45_MOESM3_ESM.tif]

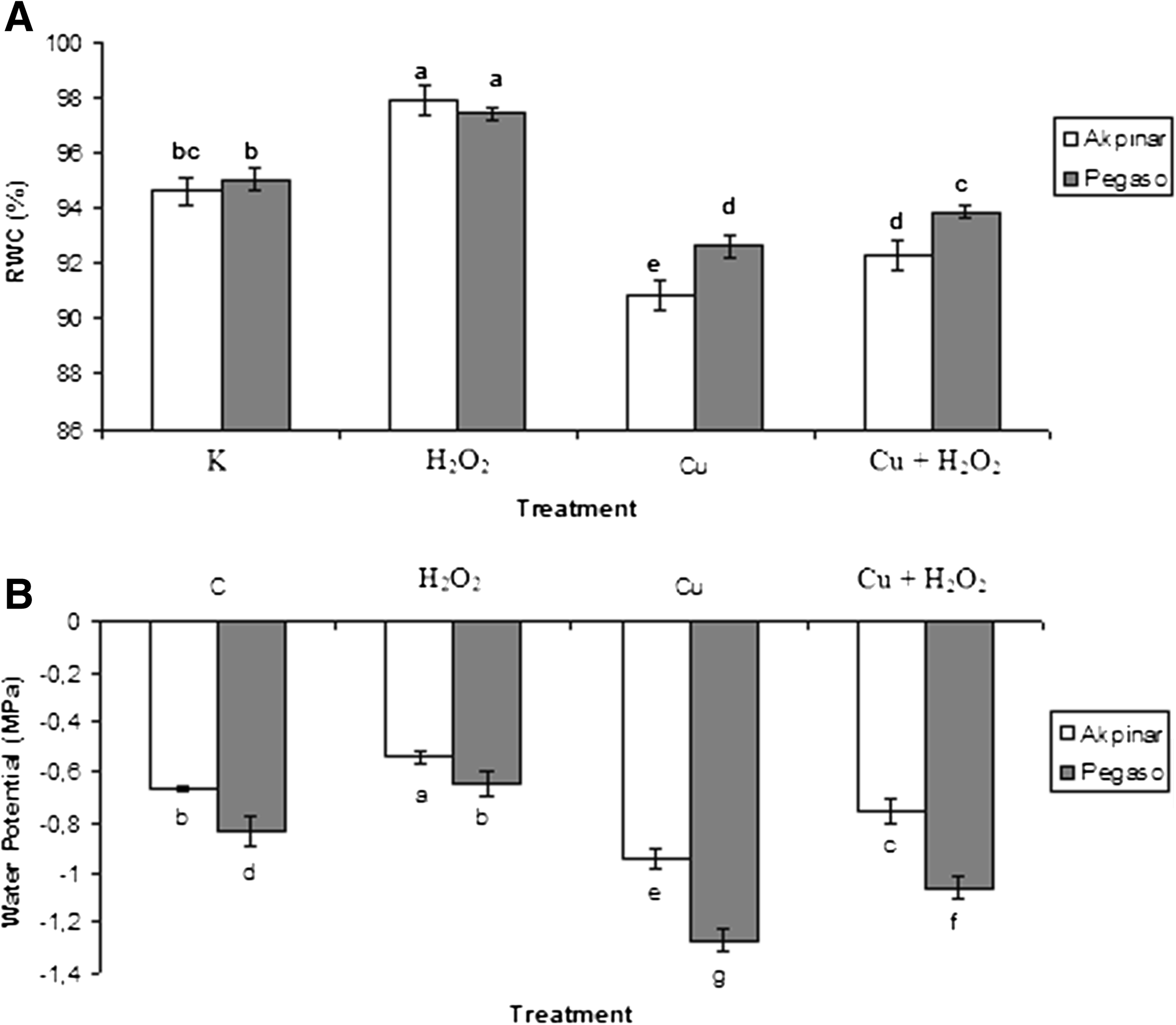

Supplement: Supplementary file 4 — Authors’ original file for figure 4 [file 40529_2012_45_MOESM4_ESM.tif]

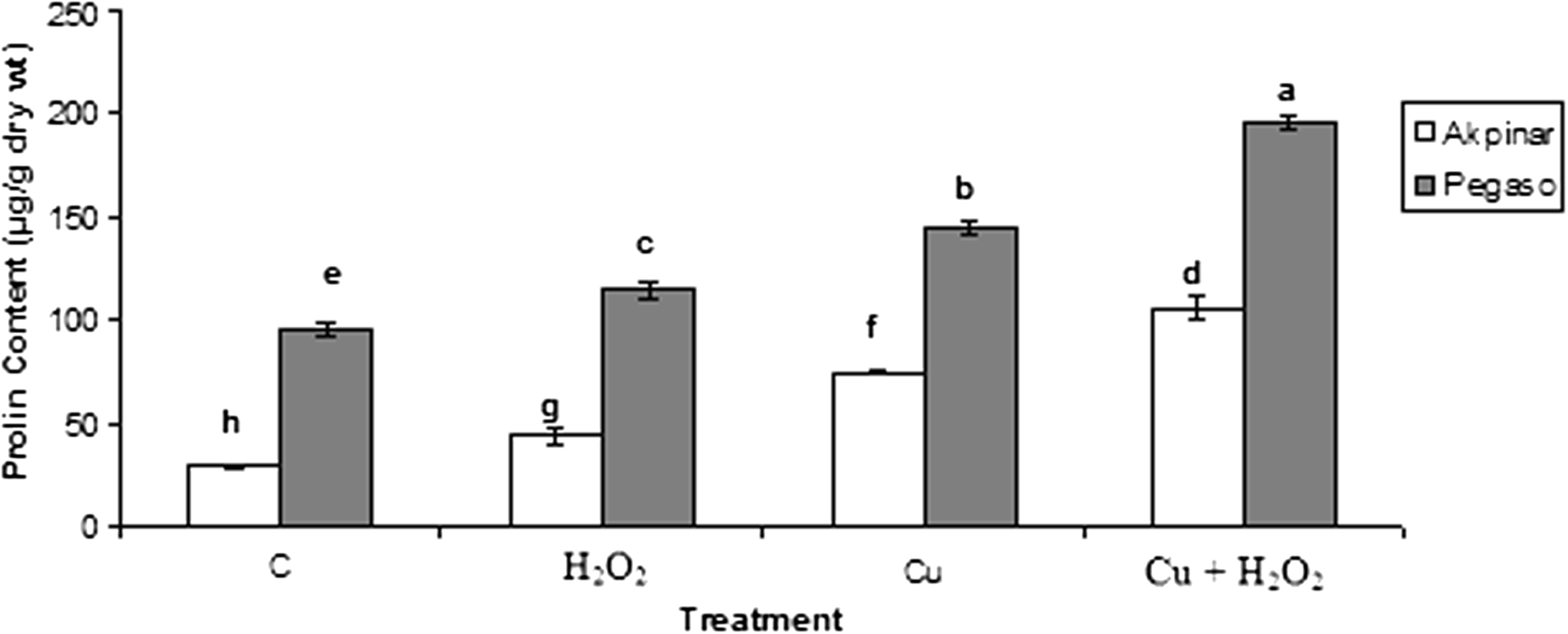

Supplement: Supplementary file 5 — Authors’ original file for figure 5 [file 40529_2012_45_MOESM5_ESM.tif]

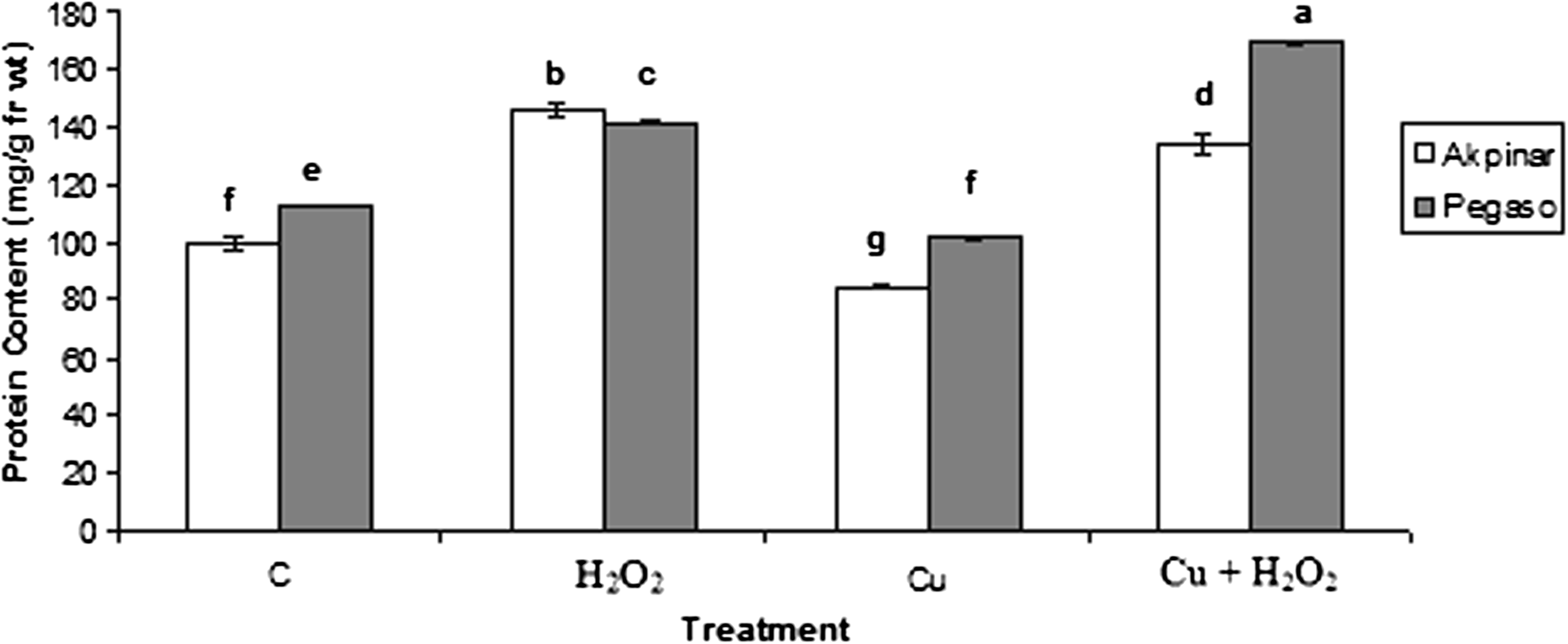

Supplement: Supplementary file 6 — Authors’ original file for figure 6 [file 40529_2012_45_MOESM6_ESM.tif]

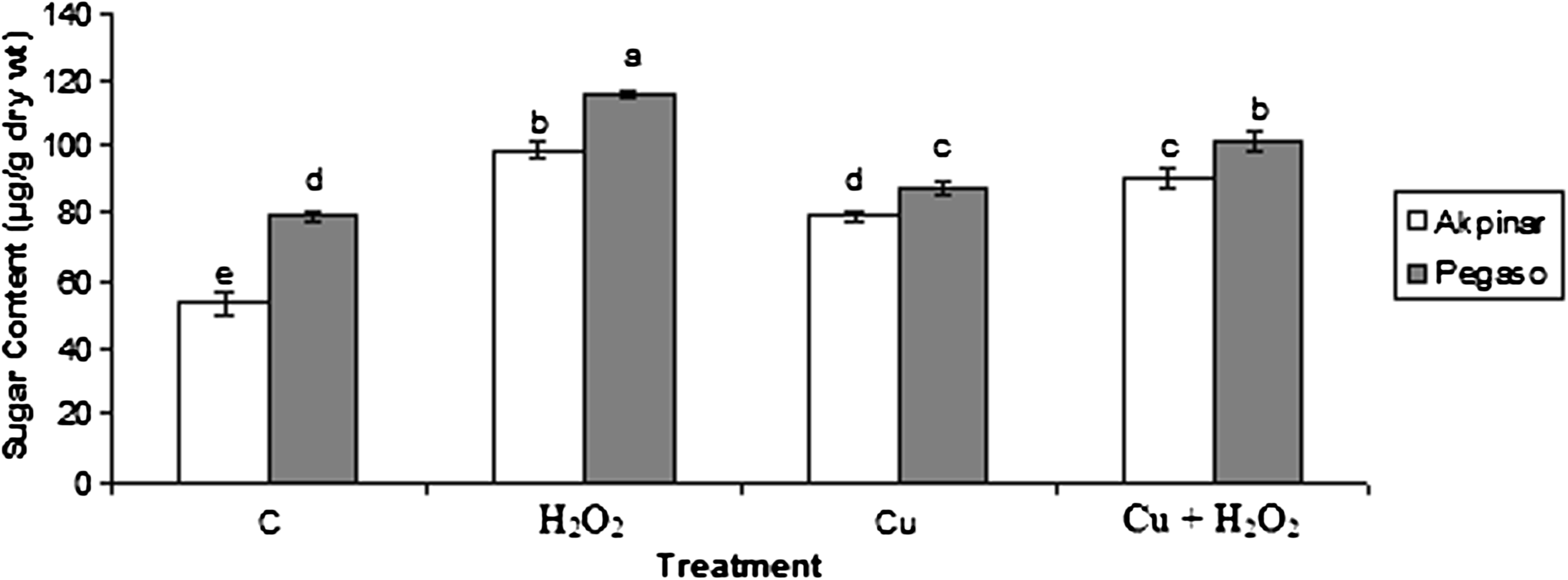

Supplement: Supplementary file 7 — Authors’ original file for figure 7 [file 40529_2012_45_MOESM7_ESM.tif]

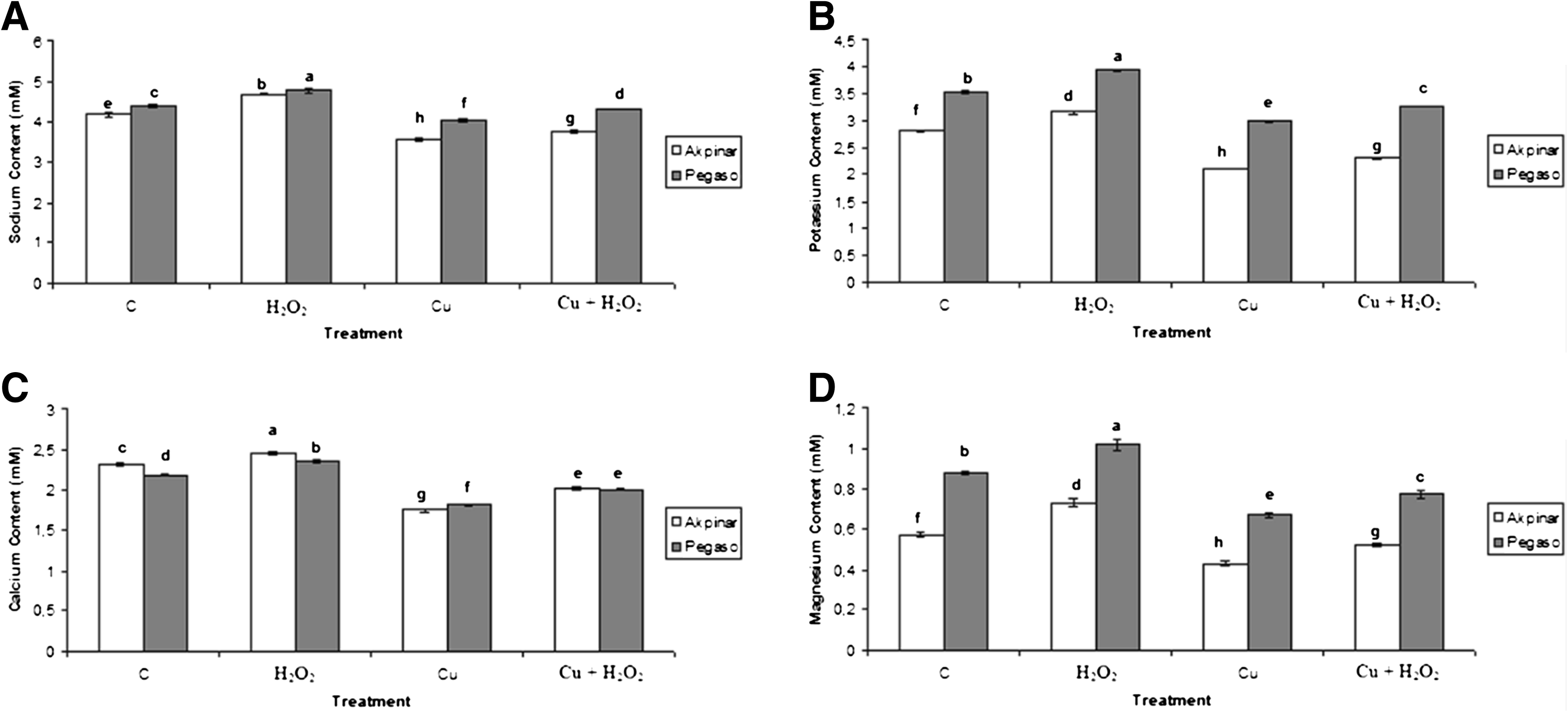

Supplement: Supplementary file 8 — Authors’ original file for figure 8 [file 40529_2012_45_MOESM8_ESM.tif]
